# Combination of Cysteine and Glutathione Prevents Ethanol-Induced Hangover and Liver Damage by Modulation of Nrf2 Signaling in HepG2 Cells and Mice

**DOI:** 10.3390/antiox12101885

**Published:** 2023-10-20

**Authors:** Hyeongyeong Kim, Hyung Joo Suh, Ki-Bae Hong, Eun-Jin Jung, Yejin Ahn

**Affiliations:** 1 Department of Integrated Biomedical and Life Science, Graduate School, Korea University, Seoul 02841, Republic of Korea; hyunkyung999@korea.ac.kr (H.K.); suh1960@korea.ac.kr (H.J.S.); 2 Transdisciplinary Major in Learning Health Systems, Department of Healthcare Sciences, Graduate School, Korea University, Seoul 02841, Republic of Korea; 3 Department of Food Science and Nutrition, Jeju National University, Jeju 63243, Republic of Korea; kbhong@jejunu.ac.kr; 4 Department of Food and Biotechnology, Korea University, Sejong 30019, Republic of Korea; ejjung1124@korea.ac.kr; 5 Research Group of Functional Food Materials, Korea Food Research Institute, Wanju-gun 55365, Republic of Korea

**Keywords:** cysteine, glutathione, ethanol, hangover, reactive oxygen species, oxidative stress, antioxidant

## Abstract

Excessive alcohol consumption increases oxidative stress, leading to alcoholic liver disease. In this study, the protective effects of a mixture of cysteine and glutathione against ethanol-induced hangover and liver damage were evaluated in mice and HepG2 cells. Ethanol (2 mL/kg) was orally administered to the mice 30 min before receiving the test compounds (200 mg/kg), and the behavioral and oxidative stress-related biochemical parameters altered by ethanol were analyzed. Acute ethanol administration increased anxiety behavior and decreased balance coordination in mice (*p* < 0.001); however, a mixture of cysteine and glutathione (MIX) in a 3:1 ratio improved alcohol-induced behavior more effectively than the individual compounds (*p* < 0.001). The MIX group showed higher ethanol-metabolizing enzyme activity than the control group (*p* < 0.001) and significantly suppressed the elevation of serum alcohol (*p* < 0.01) and acetaldehyde (*p* < 0.001) levels after 1 h of ethanol administration. In HepG2 cells, 2.5 mM MIX accelerated ethanol metabolism and reduced *cytochrome P450 2E1* mRNA expression (*p* < 0.001). MIX also increased the expression of antioxidant enzymes through the upregulation of *nuclear erythroid 2-related factor 2 (Nrf2)* signaling and consequently suppressed the overproduction of reactive oxygen species and malondialdehyde (*p* < 0.001). Collectively, MIX alleviates the hangover symptoms and attenuates the alcohol-induced oxidative stress by regulating the *Nrf2* pathway.

## 1. Introduction

The liver is the major organ that metabolizes alcohol in the human body, and chronic alcohol intake increases the risk of liver diseases, including fatty liver, hepatitis, and cirrhosis [1]. Ninety percent of alcohol absorbed through the stomach and intestines moves to the liver and is metabolized by both oxidative and non-oxidative pathways [2]. In the enzymatic oxidation pathway, ethanol is oxidized to acetaldehyde by alcohol dehydrogenase (ADH), to acetate by aldehyde dehydrogenase (ALDH), and finally excreted in the urine and breathed out as carbon-dioxide [3]. Acetaldehyde is a toxic compound that causes symptoms such as nausea, vomiting, thirst, and headache [4]. Alcohol consumers with impaired acetaldehyde metabolism have high levels of acetaldehyde, which causes liver damage, mitochondrial dysfunction, and immune system disruption [5].

Persistent and excessive alcohol consumption promotes the microsomal ethanol oxidation system rather than the dehydrogenase pathway. During this process, instead of ADH, cytochrome P450 2E1 (CYP2E1) is activated in the liver to break down acetaldehyde [6]. Ethanol metabolism by CYP2E1 generates excess reactive oxygen species (ROS) and depletes glutathione (GSH) in hepatocytes. GSH is an antioxidant that plays an important role in ROS scavenging and is present at high levels in mammalian cells. Depletion of GSH is commonly observed in chronic alcohol consumption and liver diseases involving oxidative stress [7]. Large amounts of ROS are generated by the activation of CYP2E1, which damages DNA, proteins, and lipids and accumulates lipid peroxides. The risk of alcoholic liver disease by inducing proinflammatory cytokine production and apoptosis is also increased [8]. Consequently, chronic alcohol consumption is a major contributor to oxidative stress, which not only promotes ROS production but also disrupts the redox system homeostasis of the body.

The body responds to oxidative stress through the action of antioxidant enzymes, such as superoxide dismutase (SOD), catalase (CAT) and glutathione peroxidase (GSH-Px), and non-enzymatic antioxidants [9]. In particular, the nuclear factor erythroid-2-related factor 2 (Nrf2) signaling pathway, is important for ROS scavenging via the antioxidant system. Under oxidative stress, Nrf2 dissociates from kelch-like ECH-associated protein-1 (Keap1), translocates to the nucleus, and the activated Nrf2 induces the expression of various endogenous antioxidant enzymes [10]. Nrf2 also promotes the production of heme oxygenase-1 (HO-1) to protect hepatocytes from oxidative stress, and HO-1 attenuates alcohol-induced liver damage by suppressing inflammatory responses [11]. Hence, accelerating the breakdown of alcohol and acetaldehyde and inhibiting the accumulation of ROS play important roles in protection against alcoholic liver damage.

Recently, natural antioxidants have been considered as new alternatives for relieving hangovers and treating alcoholic liver damage. The dietary intake of vitamins and amino acids also helps prevent alcoholic liver damage. L-ascorbic acid improved alcohol-induced liver damage by modulating iron metabolism in alcohol-fed mice [12]. Additionally, vitamin supplements containing L-cysteine have been reported to improve the symptoms of alcohol hangovers, such as anxiety, nausea, and headaches, in adults [13]. N-acetylcysteine was documented to attenuate alcohol-induced oxidative stress in the mouse liver [14], and methionine supplementation has been shown to exhibit antioxidant activity by upregulating the expression of antioxidant enzymes in mice [15]. These results demonstrated the biochemical properties and therapeutic functions of antioxidants and amino acids in liver diseases. However, the role of amino acid mixed with an antioxidant agent as potential therapeutic agents for liver diseases has not been studied. Therefore, this study aimed to select amino acids with antioxidant activity, and confirmed the combination of cysteine and glutathione on protection against alcohol-induced liver damage and improvement of hangover in in vitro and in vivo models.

## 2. Materials and Methods

### 2.1. Chemicals

All 16 amino acids used in the experiments were provided by Neocrema Co., Ltd. (Seoul, Republic of Korea). Also, taurine, theanine, and GSH were provided by NeoCrema. Ascorbic acid, 2,2’-azino-bis (3-ethylbenzothiazoline-6-sulfonic acid) (ABTS), and 2,2-diphenyl-1-picrylhydrazyl (DPPH) were purchased from Sigma-Aldrich (St. Louis, MO, USA).

### 2.2. Analysis of Radical Scavenging Activity of Amino Acids

ABTS and DPPH radical scavenging activities of the amino acids were analyzed as previously described [16]. The ABTS radical scavenging activity was expressed as the ascorbic acid equivalent antioxidant capacity (μg AEAC/mg) from a standard curve obtained using ascorbic acid. The concentration of the sample required to reduce the absorbance of ABTS and DPPH radicals by 50% was expressed as the 50% inhibitory concentration (IC_50_).

### 2.3. Animals and Description of Groups

Institute of Cancer Research (ICR) mice (6 weeks, male) were purchased from Orient Bio (Seongnam, Korea) and bred at the Korea University Central Laboratory Animal Center (temperature: 20–26 °C, humidity: 40–60%, illumination: 200–300 lux). Animals had access to feed (Altromin, Lage, Germany) and water ad libitum. After acclimatized for a week, the mice were randomly divided into different groups (*n* = 6 per group) as follows: NOR (normal, untreated), CON (control, 0.9% saline with ethanol), Cys (200 mg/kg Cys with ethanol), GSH (200 mg/kg GSH with ethanol), MIX (200 mg/kg mixture of Cys and GSH with ethanol). Initially, different Cys:GSH ratios were screened to determine the most appropriate ratio. To induce hangovers, 25% ethanol (2 mL/kg) was orally administered 30 min after the chemical (or 0.9% saline) administration [17].

### 2.4. Behavioral Test

Motor function and balance coordination according to ethanol intake in mice were evaluated using the balance beam test (BBT) [18]. The BBT was performed three times a day for 5 days to acclimatize and train the mice to the environment before initiating the experiments. The test was performed 0.5, 1, and 2 h after ethanol administration. Chemicals (200 mg/kg of Cys, GSH, and MIX) or 0.9% saline was pre-administered for 0.5 h before ethanol administration. The mouse was placed on a balance beam (length 100 cm, width 12 mm) located at a height of 50 cm, and the time to reach the end of the balance beam along with the number of foot slips during the movement were measured.

An elevated plus maze (EPM) is a cross-shaped maze with two closed and two open arms installed at a height of 50 cm. The anxiety-related behaviors of the mice were analyzed by the EPM test 0.5 h after ethanol intake [19]. EPM was performed 0.5 h after ethanol administration, and the samples were pre-administered 0.5 h before ethanol administration. After recording the movements of the mice for 5 min, the percentage time spent in the open arm and the number of entries into it were analyzed using the Noldus Ethovision^®^ XT (Noldus, Wageningen, The Netherlands).

### 2.5. Ethanol Metabolism Analysis

#### 2.5.1. Analysis of Ethanol-Related Biochemical Parameters in Serum

After 0.5, 1, and 2 h after ethanol administration, blood was collected from the mice’s jugular veins and centrifuged (2800× *g*, 10 min, 25 °C) to separate the serum. The alcohol and acetaldehyde contents in the serum were analyzed using an ethanol assay kit (BM-ETH-100, BIOMAX, Guri, Republic of Korea) and an aldehyde assay kit (K2096, BioVision, Milpitas, CA, USA), respectively, as per the manufacturer’s protocol. Serum levels of alanine transferase (ALT) and aspartate transaminase (AST) were measured using an automated biochemical analyzer (DRI-CHEM 3500i, Fujifilm, Co., Tokyo, Japan).

#### 2.5.2. ADH and ALDH Activities in Liver

After 2 h of ethanol administration, the experimental animals were sacrificed under CO2 anesthesia, and liver tissues were collected. After washing three times with PBS, the liver tissues were homogenized by adding 0.1 M Tris-HCl buffer (pH 7.4) in a volume 10 times the tissue weight. ADH and ALDH activities were measured using the ADH assay kit (#ab102533, Abcam, Cambridge, UK) and the ALDH activity assay kit (#ab155893, Abcam), respectively.

### 2.6. ROS and Malondialdehyde (MDA) Contents

Liver tissues (50 mg) were homogenized by adding 40 mM Tris-HCl buffer and centrifuged (12,000× *g*, 10 min, 4 °C). The obtained supernatants (50 μL) were reacted at 37 °C for 30 min in the dark after adding 40 mM Tris-HCl buffer (450 μL) and 10 μM 2′,7′-dichlorofluorescein diacetate (10 μL). The ROS content in the tissues was measured by fluorescence analysis (excitation, 482 nm; emission, 535 nm). Additionally, the MDA content was analyzed using the OxiTec™ TBARS assay kit (#BO-TBR-200, BIOMAX), according to the manufacturer’s protocol.

### 2.7. Cell Culture and Viability Assay

Human hepatoblastoma (HepG2) cells were purchased from the Korea Cell Line Bank (Seoul, Republic of Korea). The cells were cultured in a CO2 incubator (5% CO_2_, 37 °C) using Dulbecco’s modified Eagle’s medium supplemented with 10% fetal bovine serum and 1% penicillin-streptomycin. To determine the effect of the amino acid treatment on cell viability, HepG2 cells were seeded in 96-well plates at a density of 1 × 10^5^ cells/well and cultured for 24 h. After treatment with various concentrations of Cys, GSH, and MIX (Cys:GSH = 3:1) for 24 h, the cell viability was measured using a WST-8 assay kit (BIOMAX) as per the manufacturer’s instructions. In addition, chemicals (Cys, GSH, and MIX) were treated in the presence or absence of ethanol (700 mM) for 24 h to confirm the protective effect of chemicals against ethanol-induced cytotoxicity.

### 2.8. Quantitative Real-Time Polymerase Chain Reaction (qRT-PCR)

Two concentrations (1.0 and 2.5 mM) of Cys (CL and CH groups, respectively), GSH (GL and GH groups, respectively), and MIX (ML and MH groups, respectively) were tested in HepG2 cells. Untreated cells are indicated as UTC and ethanol-treated cells as ETC. Total RNA from the liver tissue (100 mg) and HepG2 cells were extracted using TRIzol reagent (#15596026, Invitrogen, Waltham, MA, USA) according to a previously described method [20]. The mRNA expression of the target genes was analyzed using SYBR™ Green PCR Master Mix (#4309155, Applied Biosystems, Foster City, CA, USA), and *glyceraldehyde-3-phosphate dehydrogenase (GAPDH)* was used as an endogenous gene. Target genes used in the mouse model were *CAT* (NM_009804.2), *GSH-Px* (NM_001329527.1), *SOD1* (NM_011434.2), and *GAPDH* (NM_001289726.2). In addition, *CAT* (NM_001752.4), *SOD1* (NM_000454.4), *Gpx* (NM_000581.4), ADH (NM_001042765.1), *ALDH* (NM_000689.4), *CYP2E1* (NM_000773.4), *Nrf2* (NM_001145412.3), *Keap 1* (NM_012289.4), *HO-1* (NM_002133.2), and *GAPDH* (NM_001256799.3) were used as target genes in the HepG2 cells.

### 2.9. Western Blot Analysis

HepG2 cells were seeded into a 6-well plate at 1 × 10^5^ cells/mL and cultured for 24 h. Hepg2 cells were incubated with chemicals (Cys, GSH, and MIX) for 2 h and then treated with ethanol (700 mM) for 24 h. Proteins were extracted using RIPA buffer (#ab288006, Abcam). Fifty micrograms of protein were loaded onto a 10% polyacrylamide gel, electrophoresed (135 V, 1.5 h), and transferred (40 V, 16 h) to a polyvinylidene difluoride membrane. The primary antibodies (Nrf2 [#23832], Keap1 [#4678], HO-1 [#5853]) were reacted at 4 °C for 16 h, and the secondary antibody (anti-rabbit IgG, #7074) was reacted at 25 °C for 2 h. Next, the membrane was incubated with electrochemiluminescence substrate (#1705060, Bio-Rad, Hercules, CA, USA) for 5 min and analyzed using FluorChem M (ProteinSimple, Santa Clara, CA, USA). All antibodies were purchased from Cell Signaling Technology (Danvers, MA, USA) and diluted in 5% skim milk according to the manufacturer’s protocol.

### 2.10. Statistical Analysis

Data are expressed as mean ± standard error (SEM) for the in vivo studies and mean ± standard deviation (SD) for the in vitro studies. All data were statistically processed using SPSS (Version 25.0, IBM, Chicago, IL, USA); one-way analysis of variance (ANOVA) was performed, and post hoc verification was confirmed by Tukey’s test at *p* < 0.05.

## 3. Results

### 3.1. Radical Scavenging Activity of Amino Acids

The ABTS radical scavenging activity was analyzed to determine the antioxidant activity of the amino acids (Table S1). Among the 16 amino acids, Cys showed the highest antioxidant activity with 870.84 μg AEAC/mg, followed by arginine (4.43 μg AEAC/mg) and histidine (3.81 μg AEAC/mg). The ABTS and DPPH radical scavenging activities of taurine, theanine, and GSH, along with those of the amino acids (Cys, arginine, and histidine), are shown in Table 1. In ABTS radical analysis, Cys (0.05 mg/mL) and GSH (0.29 mg/mL) displayed significantly lower IC_50_ values than other amino acids (*p* < 0.05). Similarly, in the DPPH radical analysis, the IC_50_ values of the two chemicals (0.05 and 10.80 mg/mL, respectively) were significantly lower than those of other amino acids (*p* < 0.05). Therefore, Cys and GSH with high antioxidant activity were selected and subsequent studies were undertaken wherein the hangover-relieving effects of the individual and mixed amino acids were confirmed in in vitro and in vivo models.

### 3.2. Effects of Cys, GSH, and MIX on Alcohol-Induced Behavioral Changes

**Figure 1:**
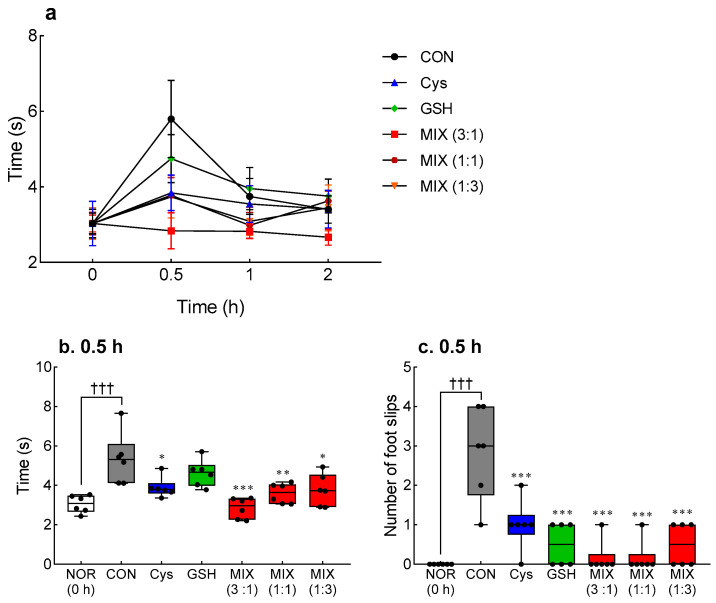
Effects of Cys, GSH, and MIX treatment on motor coordination analyzed with the balance beam test in ethanol-treated ICR mice. (**a**) Balance beam reaching time over time after ethanol treatment, (**b**) Balance beam reaching time and (**c**) number of foot slips after 0.5 h of ethanol treatment. Cys, GSH, and MIX were orally pretreated 30 min before administration of ethanol (2 mL/kg). The balance beam test was measured before and 0.5, 1, and 2 h after ethanol intake. NOR: untreated group (0 h), CON: ethanol-treated group, Cys: 200 mg/kg Cys-treated group, GSH: 200 mg/kg GSH-treated group, MIX: 200 mg/kg MIX-treated group. Values represent mean ± SEM. ^†††^ *p* < 0.001 vs. NOR (0 h) group; * *p* < 0.05, ** *p* < 0.01, and *** *p* < 0.001 vs. CON group (ANOVA followed by Tukey’s test). Cys, cysteine; GSH, glutathione; MIX, the mixture of Cys and GSH in ratios of 3:1, 1:1, and 1:3.

To select the appropriate mixing ratio of Cys and GSH, changes in alcoholic behavior followed by individual and mixed treatments of Cys and GSH were confirmed using the BBT (Figure 1). The BBT was performed at 0.5, 1, and 2 h after alcohol administration, and differences between the experimental groups were observed at 0.5 h (Figure 1a). After alcohol administration (0.5 h), the total reach time (*p* < 0.001, Figure 1b) and the number of foot slips (*p* < 0.001, Figure 1c) were significantly increased in the control group (CON) compared with those before alcohol administration. The Cys-treated group (3.8 s, *p* < 0.05) had a significantly reduced arrival time compared to the CON group (5.8 s), and the GSH-treated group (4.7 s) displayed a decreasing tendency (Figure 1b). The MIX-treated groups had significantly shorter reach times than the CON group at all ratios tested (3:1, *p* < 0.001; 1:1, *p* < 0.01; and 1:3, *p* < 0.01). In particular, MIX treatment (3:1) showed a shorter reach time (2.8 s) than individual chemical treatments (Cys and GSH). The number of foot slips which increased by alcohol administration was also significantly improved by individual and mixed treatment of Cys and GSH administration (*p* < 0.001, Figure 1b), with the MIX-treated (3:1) group showing the lowest value. Overall, alcohol-induced behaviors in the MIX-treated group were effectively improved at a 3:1 ratio; therefore, this ratio of Cys to GSH was deemed to be most appropriate and used in further experiments.

**Figure 2:**
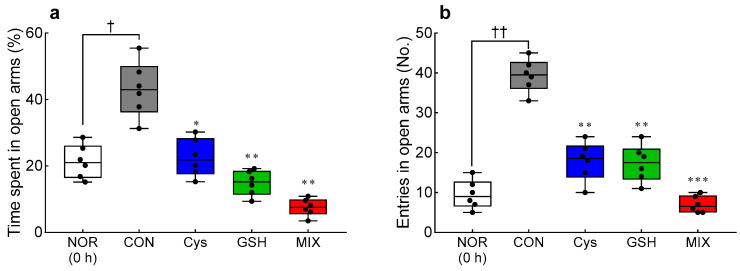
Effects of Cys, GSH, and MIX treatment on anxiety-related behavior analyzed with the elevated plus maze test in ethanol-treated ICR mice. After 0.5 h of ethanol treatment, (**a**) % time spent in the open arm and (**b**) number of entries. Cys, GSH, and MIX were orally pretreated 30 min before administration of ethanol (2 mL/kg). The elevated plus maze test was performed before ethanol intake (0 h) and 0.5 h after ethanol intake. NOR: untreated group (0 h), CON: ethanol-treated group, NOR: untreated group (0 h), CON: ethanol-treated group, Cys: 200 mg/kg Cys-treated group, GSH: 200 mg/kg GSH-treated group, MIX: 200 mg/kg MIX-treated group. Values represent mean ± SEM. Different symbols indicate statistically significant differences at the *p* < 0.05 level (ANOVA followed by Tukey’s test): ^†^ *p* < 0.05, ^††^ *p* < 0.01 vs. NOR (0 h) group; * *p* < 0.05, ** *p* < 0.01, and *** *p* < 0.001 vs. CON group. Cys, cysteine; GSH, glutathione; MIX, the mixture of cysteine and glutathione in a 3:1 ratio.

The alcoholic anxiety behaviors of individuals and mixed treatment of Cys and GSH were confirmed using the EPM (Figure 2). After ethanol administration (0.5 h), the % of time spent in the open arm (*p* < 0.05, Figure 2a) and the number of entries (*p* < 0.01, Figure 2b) increased significantly in the CON group compared to those at 0 h. Time spent in the open arms was significantly reduced in the Cys-treated (21%, *p* < 0.05), GSH-treated (12%, *p* < 0.01), and MIX-treated (3%, *p* < 0.01) groups compared to that in the CON (45%) group. As compared to the number in the CON group, the number of open-arm entries was also significantly lower in the Cys-treated (0.42-fold, *p* < 0.01), GSH-treated (0.38-fold, *p* < 0.01), and MIX-treated (0.14-fold, *p* < 0.001) groups. In particular, the MIX administration reduced the time spent in the open arm and the number of entries more effectively than the individual chemicals.

### 3.3. Effects of Cys, GSH, and MIX on Alcohol Metabolism-Related Factors in Serum and Liver Tissue

**Figure 3:**
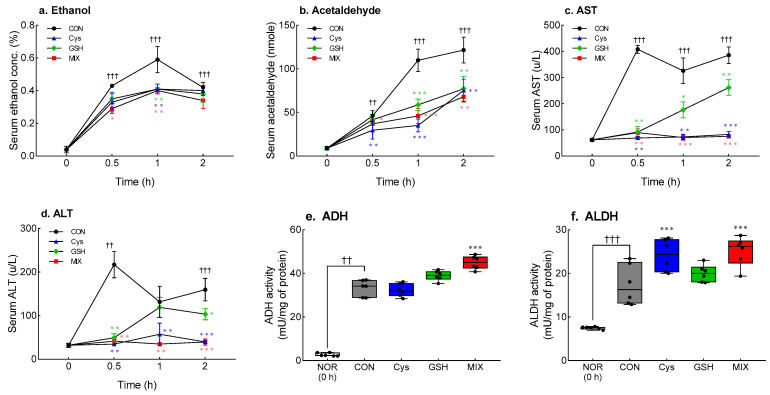
Effects of Cys, GSH, and MIX treatment on ethanol metabolism-related factors in the serum and liver of ethanol-treated ICR mice. Cys, GSH, and MIX were orally pretreated 30 min before administration of ethanol (2 mL/kg). After 0.5, 1, and 2 h of ethanol administration, blood was collected, and biochemical analysis was performed. In addition, after 2 h of ethanol administration, the animals were sacrificed and ADH and ALDH activities were analyzed in liver tissue. NOR: untreated group (0 h), CON: ethanol-treated group, Cys: 200 mg/kg Cys-treated group, GSH: 200 mg/kg GSH-treated group, MIX: 200 mg/kg MIX-treated group. Values represent mean ± SEM. Different symbols indicate statistically significant differences at *p* < 0.05 (ANOVA followed by Tukey’s test): ^††^ *p* < 0.01, ^†††^ *p* < 0.001 vs. NOR (0 h) group; * *p* < 0.05, ** *p* < 0.01, and *** *p* < 0.001 vs. CON group. Cys, cysteine; GSH, glutathione; MIX, the mixture of cysteine and glutathione in a 3:1 ratio. AST, aspartate transaminase; ALT, alanine transferase; ADH, alcohol dehydrogenase; ALDH, aldehyde dehydrogenase.

Changes in the serum concentrations of alcohol and acetaldehyde over time after ethanol administration are shown in Figure 3a,b. Serum ethanol concentration reached its highest level in all experimental groups 1 h after ethanol administration and gradually decreased thereafter (Figure 3a). Alcohol concentration was significantly reduced by 67.4% in the MIX-treated group compared to that in the CON group after 0.5 h of ethanol administration (*p* < 0.05). In addition, 1 h after ethanol administration, the Cys-treated, the GSH-treated, and the MIX-treated groups showed a significant decrease in alcohol concentration compared to that in the CON group (*p* < 0.01). The concentration of acetaldehyde in the blood exhibited a tendency to gradually increase with increasing ethanol administration time in all the experimental groups (Figure 3b). One hour after alcohol administration, the concentrations of acetaldehyde in the three treatment groups were significantly lower than those in the CON group (*p* < 0.001). In addition, 2 h after alcohol administration, the concentration of acetaldehyde decreased significantly in the following order: Cys-treated (0.61-fold), GSH-treated (0.63-fold), and MIX-treated (0.56-fold) compared to that in the CON group (*p* < 0.01).

Figure 3c,d depict the changes in serum AST and ALT levels over time after ethanol administration. Serum AST (*p* < 0.01, Figure 3c) and ALT (*p* < 0.05, Figure 3d) levels in the CON group were significantly increased after ethanol administration (0.5 h) compared to 0 h. Administration of Cys, GSH, and MIX significantly suppressed the rapid increase in AST (*p* < 0.01) and ALT (*p* < 0.01) levels 0.5 h after ethanol administration. In particular, the MIX-treated group showed the lowest AST and ALT levels among all experimental groups; the levels being equivalent to those before alcohol administration.

The effects of individual and mixed treatment of Cys and GSH on alcohol metabolism were measured as ADH and ALDH activities in the liver tissue 2 h after ethanol administration (Figure 3e,f). The CON group showed significantly higher ADH (*p* < 0.01, Figure 3e) and ALDH (*p* < 0.001, Figure 3f) activity than before ethanol administration. MIX administration markedly increased ADH (*p* < 0.001) and ALDH (*p* < 0.001) activities by 1.35 times and 1.34 times, respectively, compared to those in the CON group. The Cys-treated group also showed 1.30 times higher ALDH activity than the CON group (*p* < 0.001).

### 3.4. Effects of Cys, GSH, and MIX on Oxidative Stress-Related Factors in the Liver

**Figure 4:**
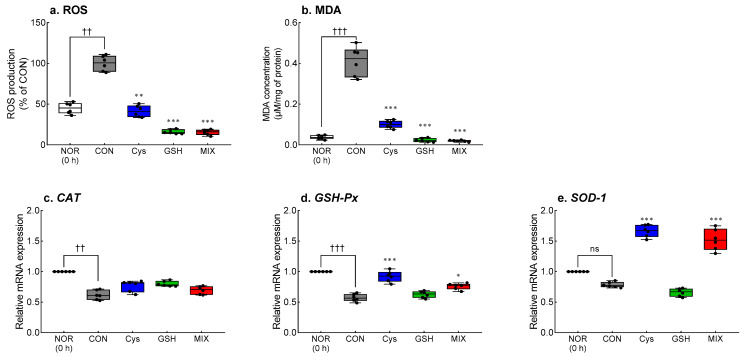
Effects of Cys, GSH, and MIX treatment on (**a**) ROS and (**b**) MDA contents, and (**c**–**e**) antioxidant-related mRNA expression in the liver of ethanol-treated ICR mice. Cys, GSH, and MIX were orally pretreated 30 min before administration of ethanol (2 mL/kg). After 2 h of ethanol administration, animals were sacrificed, and biochemical analysis of liver tissue was performed. NOR: untreated group (0 h), CON: ethanol-treated group, Cys: 200 mg/kg Cys-treated group, GSH: 200 mg/kg GSH-treated group, MIX: 200 mg/kg MIX-treated group. Values represent mean ± SEM. Different symbols indicate statistically significant differences at *p* < 0.05 (ANOVA followed by Tukey’s test): ^††^ *p* < 0.01, ^†††^ *p* < 0.001 vs. NOR group; *p < 0.05, ** *p* < 0.01, and *** *p* < 0.001 vs. CON group. Cys, cysteine; GSH, glutathione; MIX, the mixture of cysteine and glutathione in a 3:1 ratio; ROS, reactive oxygen species; MDA, malondialdehyde; CAT, catalase; GSH-Px, glutathione peroxidase; SOD-1, superoxide dismutase-1; ns, not significant.

Ethanol administration significantly promoted the accumulation of ROS (*p* < 0.01, Figure 4a) and MDA (*p* < 0.01, Figure 4b) in the liver. Administration of Cys, GSH, and MIX significantly suppressed the production of ROS and MDA (*p* < 0.01 and *p* < 0.001, respectively). In particular, the MIX-treated group had the lowest ROS and MDA levels among the experimental groups. Acute ethanol administration significantly reduced the mRNA expression of *CAT* (*p* < 0.01, Figure 4c) and *GSH-Px* (*p* < 0.001, Figure 4d). The expression of *SOD-1* tended to decrease in the CON group compared to that before ethanol administration, but the difference was not significant (Figure 4e). The Cys-treated and MIX-treated groups showed significantly higher expression of *GSH-Px* (*p* < 0.05 and *p* < 0.001, respectively) and *SOD-1* (*p* < 0.001) than that in the CON group.

### 3.5. Effects of Cys, GSH, and MIX on Cell Viability in HepG2 Cells

**Figure 5:**
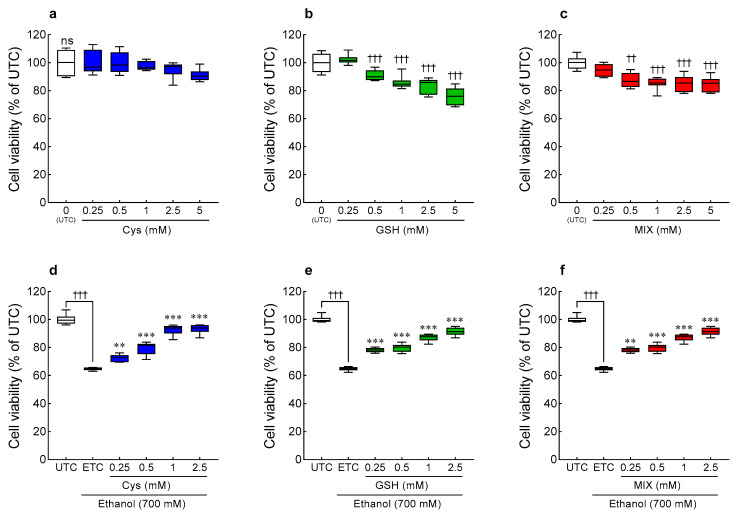
Effect of Cys, GSH, and MIX treatment on cell viability in the absence (**a**–**c**) and presence (**d**–**f**) of ethanol in HepG2 cells. HepG2 cells were treated with Cys, GSH, and MIX in the presence or absence of ethanol for 24 h. UTC: untreated cells, ETC group: ethanol-treated cells. Values are mean ± SD. Different symbols indicate significant differences at the *p* < 0.05 level (ANOVA followed by Tukey’s test). ^††^ *p* < 0.01, ^†††^ *p* < 0.001 vs. UTC group; ** *p* < 0.01 and ***p < 0.001 vs. ETC group. ns, not significant. Cys, cysteine; GSH, glutathione; MIX, the mixture of cysteine and glutathione in a 3:1 ratio.

The effect of individual and mixed treatment of Cys and GSH on the proliferation of HepG2 cells was confirmed using the WST assay (Figure 5a–c). Following treatment with different concentrations of Cys (Figure 5a), there was no significant difference when compared with the untreated cells (UTC). GSH and MIX showed a significant difference from the UTC at concentrations of 0.5 mM and higher (*p* < 0.01, and *p* < 0.001, respectively), and cell viability was above 80% at all concentrations (Figure 5b,c). Thus, Cys (95.1–100.1%), GSH (85.1–94.7%), and MIX (83.5–102.3%) showed no cytotoxicity in HepG2 cells at concentrations below 2.5 mM.

The hepatocellular protective effects of individual and mixed treatment of Cys and GSH were confirmed in HepG2 cells subjected to ethanol-induced oxidative stress (Figure 5d–f). Ethanol treatment significantly reduced the viability of HepG2 cells to 64.8% (*p* < 0.001). However, treatment with Cys, GSH, and MIX significantly increased the cell viability compared to the ETC in a dose-dependent manner (*p* < 0.01 and *p* < 0.001, respectively). In particular, cell viability was decreased by ethanol and was restored to a level equivalent to that of the UTC at a concentration of 1 mM or higher. Therefore, treatment with Cys, GSH, or MIX protects against ethanol-induced cytotoxicity.

### 3.6. Effects of Cys, GSH, and MIX on Ethanol Metabolism-Related Factors in HepG2 Cells

**Figure 6:**
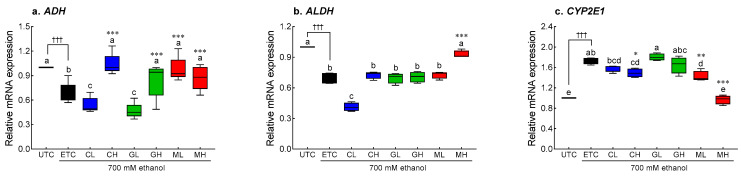
Effects of Cys, GSH, and MIX treatment on alcohol metabolism-related mRNA expression in ethanol-treated HepG2 cells. HepG2 cells were treated with Cys, GSH, and MIX with 700 mM ethanol for 24 h. UTC: untreated cells, ETC: ethanol-treated cells, CL: 1.0 mM Cys treated cells, CH: 2.5 mM Cys treated cells, GL: 1.0 mM GSH treated cells, GH: 2.5 mM GSH treated cells, ML: 1.0 mM MIX treated cells, MH: 2.5 mM MIX treated cells. Values are mean ± SD. Different letters indicate significant differences at the *p* < 0.05 level (ANOVA followed by Tukey’s test). ††† *p* < 0.001 vs. NOR group; * *p* < 0.05, ** *p* < 0.01 and *** *p* < 0.001 vs. ETC. Cys, cysteine; GSH, glutathione; MIX, the mixture of cysteine and glutathione in a 3:1 ratio; *ADH*, alcohol dehydrogenase; *ALDH*, aldehyde dehydrogenase; *CYP2E1*, cytochrome P450 family 2 subfamily E member 1.

Ethanol treatment significantly reduced *ADH* (0.62-fold, *p* < 0.001) and *ALDH* (0.69-fold, *p* < 0.001) mRNA expression in HepG2 cells (Figure 6a,b). Treatment with Cys and GSH (2.5 mM) significantly increased *ADH* expression compared to that in the ETC (*p* < 0.001, Figure 6a). As compared to the ETC, the *ADH* expression was significantly higher in cells treated with MIX (*p* < 0.001, Figure 6a). *ALDH* expression was also significantly increased by high-dose MIX treatment (*p* < 0.001, Figure 6b). In contrast, ethanol treatment significantly increased *CYP2E1* expression by 1.73 times (*p* < 0.001, Figure 6c). High-dose Cys treatment significantly reduced *CYP2E1* expression compared to the ETC (*p* < 0.05), and MIX treatment significantly reduced *CYP2E1* expression in a dose-dependent manner (*p* < 0.01 and *p* < 0.001, respectively). In particular, 2.5 mM MIX treatment restored the expression of *CYP2E1* to a level similar to that observed in the UTC.

### 3.7. Effects of Cys, GSH, and MIX on Oxidative Stress-Related Factors in HepG2 Cells

**Figure 7:**
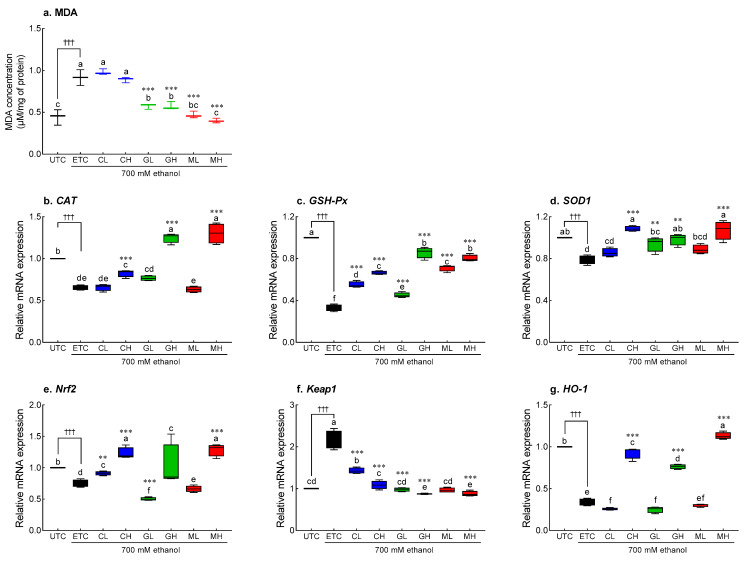
Effects of Cys, GSH, and MIX treatment on oxidative stress-related mRNA expression in ethanol-treated HepG2 cells. HepG2 cells were treated with Cys, GSH, and MIX with 700 mM ethanol for 24 h. UTC: untreated cells, ETC: ethanol-treated cells, CL: 1.0 mM Cys treated cells, CH: 2.5 mM Cys treated cells, GL: 1.0 mM GSH treated cells, GH: 2.5 mM GSH treated cells, ML: 1.0 mM MIX treated cells, MH: 2.5 mM MIX treated cells. Values are mean ± SD. Different letters indicate significant differences at the *p* < 0.05 level (ANOVA followed by Tukey’s test). ^†††^ *p* < 0.001 vs. UTC group; ** *p* < 0.01 and *** *p* < 0.001 vs. ETC group. Cys, cysteine; GSH, glutathione; MIX, mixture of cysteine and glutathione in a 3:1 ratio; MDA, malondialdehyde; CAT, catalase; GSH-Px, glutathione peroxidase; SOD1, superoxide dismutase 1; Nrf2, nuclear factor erythroid 2–related factor 2; Keap1, kelch-like ECH-associated protein 1; HO-1, heme oxygenase-1.

Ethanol treatment increased MDA accumulation 2.5 times (*p* < 0.001), whereas GSH and MIX treatment significantly inhibited MDA production (*p* < 0.001, Figure 7a). In addition, ethanol treatment significantly decreased mRNA expression of antioxidant enzymes including *CAT* (0.65-fold; Figure 7b), *GSH-Px* (0.33-fold, Figure 7c), and *SOD-1* (0.79-fold, Figure 7d) compared to UTC (*p* < 0.001). High-dose Cys, GSH, and MIX treatment significantly increased the mRNA expression of *CAT* (*p* < 0.01 and *p* < 0.001, respectively) and *SOD-1* (*p* < 0.01 and *p* < 0.001, respectively) compared to the ETC. The expression of *GSH-Px* increased in a dose-dependent manner following Cys, GSH, and MIX treatment (*p* < 0.001). Ethanol significantly suppressed the expression of *Nrf2* (*p* < 0.001, Figure 7e) and *HO-1* (*p* < 0.001, Figure 7g) more than UTC, but increased the expression of *Keap1* (Figure 7f, *p* < 0.001).

**Figure 8:**
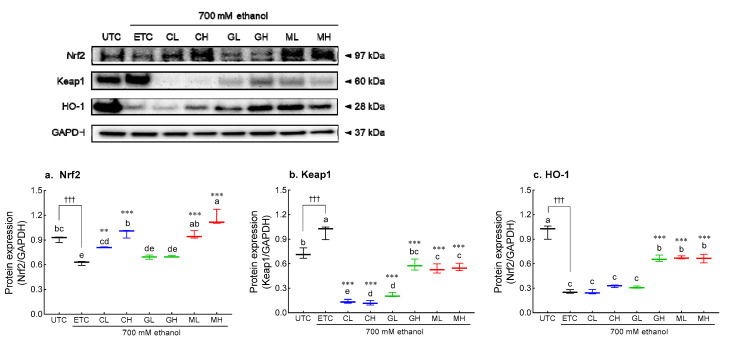
Effects of CYS, GSH, and MIX treatment on Nrf2/Keap1 pathway-related protein expression in ethanol-treated HepG2 cells. HepG2 cells were treated with Cys, GSH, and MIX with 700 mM ethanol for 24 h. UTS: untreated cells, ETS: ethanol-treated cells, CL: 1.0 mM Cys treated cells, CH: 2.5 mM Cys treated cells, GL: 1.0 mM GSH treated cells, GH: 2.5 mM GSH treated cells, ML: 1.0 mM MIX treated cells, MH: 2.5 mM MIX treated cells. Values are mean ± SD. Different letters indicate significant differences at the *p* < 0.05 level (ANOVA followed by Tukey’s test): ††† *p* < 0.001 vs. NOR group; ** *p* < 0.01 and *** *p* < 0.001 vs. CON group. Cys, cysteine; GSH, glutathione; MIX, mixture of cysteine and glutathione in a 3:1 ratio; Nrf2, nuclear factor erythroid 2–related factor 2; Keap1, kelch-like ECH-associated protein 1; HO-1, heme oxygenase-1.

The effects of Cys, GSH, and MIX on Nrf2 signaling in ethanol-treated HepG2 cells were evaluated by qRT-PCR (Figure 7e–g) and Western blotting (Figure 8). Ethanol treatment significantly suppressed the expression of *Nrf2* (*p* < 0.01 and *p* < 0.001, respectively) and *HO-1* (*p* < 0.001), but significantly increased the expression of Keap1 (*p* < 0.01 and *p* < 0.001, respectively). Likewise, Cys treatment significantly increased the mRNA expression of *Nrf2* (*p* < 0.01 and *p* < 0.001, Figure 7e) *and HO-1* (*p* < 0.001, Figure 7g), and suppressed the expression of Keap1 (*p* < 0.001, Figure 7f) in a dose-dependent manner. GSH treatment significantly decreased the expression of *Keap1* (*p* < 0.001) compared to the ETC; in particular, the high-dose GSH treatment increased the expression of *HO-1* (*p* < 0.001). The treatment with 2.5 mM MIX significantly increased the mRNA expression of *Nrf2* and *HO-1* compared to that in the CON group (*p* < 0.001), which was higher than that in the Cys-treated and GSH-treated groups. The mRNA expression of *Keap1* (*p* < 0.001) was significantly decreased by MIX treatment in a dose-dependent manner.

Similarly, MIX treatment significantly restored the protein expression of Nrf2 (*p* < 0.001, Figure 8a) and HO-1 (*p* < 0.001, Figure 8c) that had been reduced by ethanol. Cys-treated groups had significantly higher Nrf2 protein expression than the ETC group in a dose-dependent manner (*p* < 0.01 and *p* < 0.001, respectively). Treatment with 2.5 mM GSH significantly increased HO-1 protein expression (*p* < 0.001). In addition, the Cys-treated, GSH-treated, and MIX-treated groups showed significantly lower Keap1 protein expression than that in the ETC (*p* < 0.001, Figure 8b).

## 4. Discussion

In modern society, alcohol has long been consumed to form social relationships and relieve stress. However, alcohol-related diseases and accidents caused due to excessive drinking increase the socioeconomic burden worldwide [21]. Alcohol depresses the central nervous system and causes neurological and behavioral changes. Moderate drinking relieves tension and improves mood, but high blood ethanol levels lead to loss of self-control and behavioral control, increased excitability and aggression, and reduced motor coordination [22]. A study showed that repeated and prolonged alcohol administration increases depressive and anxious behaviors in mice, which are associated with reduced iron homeostasis and synaptic plasticity [23]. King et al. [24] reported that chronic alcohol-treated mice exhibit memory-deficient behavior, increased liver damage, and hippocampal neuroinflammation. In this study, acute alcohol administration decreased motor coordination and increased anxious behavior in mice, whereas the administration of mixed treatment of Cys and GSH effectively improved alcohol-related behavior (Figure 1 and Figure 2).

The liver metabolizes ethanol through an enzymatic pathway driven primarily by ADH, which oxidizes acetaldehyde. Chronic alcohol consumption causes hepatic mitochondrial dysfunction and reduces ALDH activity, leading to acetaldehyde accumulation [25]. Acetaldehyde causes alcoholic liver damage, such as fatty liver and hepatic fibrosis, by accelerating the production of free radicals and the accumulation of lipid peroxides [26]. ADH and ALDH activities vary from person to person, and the Asian population have a higher genetic deficiency in ALDH2 than those in the West [27]. ALDH2-deficient mice are susceptible to alcohol-induced liver inflammation [28]. Therefore, the increased activities of ADH and ALDH promote ethanol metabolism and help rapidly eliminate toxic metabolites. Xiao et al. [29] demonstrated that chicken-derived enzymatic hydrolysates containing essential amino acids prevented acute liver injury in mice by increasing the activity of alcohol-metabolizing enzymes. When the liver is damaged, large amounts of transaminases flow into the blood, increasing the concentrations of AST and ALT. Elevated serum ALT levels are observed in acute hepatitis, and the ratio of AST to ALT is an important indicator of alcoholic hepatitis [30]. Wang et al. [11] reported that the peduncles of *Hovenia dulcis* protected against acute alcohol-induced liver damage through radical scavenging activity and prevented the elevation of serum AST and ALT levels. In this study, the mixed amino acids accelerated alcohol metabolism, rapidly breaking down alcohol and acetaldehyde, and prevented liver damage by preventing leakage of AST and ALT (Figure 3).

The liver is vulnerable to damage from the oxidative stress caused by alcohol metabolism. High blood alcohol concentrations activate the CYP2E1-induced oxidative pathway, resulting in the overproduction of ROS and the depletion of endogenous antioxidants [31]. Lu et al. [32] found that ethanol-induced fatty liver and cirrhosis in wild-type and humanized CYP2E1 knock-in mice but attenuated ethanol-induced fatty liver in CYP2E1 knockout mice. These results suggest that CYP2E1 plays an important role in ethanol-induced fatty liver disease and oxidative stress. The present study showed that mixed amino acids inhibited CYP2E1 activation and promoted ethanol metabolism in ethanol-treated HepG2 cells (Figure 6). Oxidative stress in the liver is regulated by both enzymatic and nonenzymatic antioxidant systems [33]. In particular, sulfur-containing compounds exist in thiol groups (-SH) or oxidized forms (-S-) in biomolecules and protect against radical-induced damage by regulating redox reactions. Cys is a sulfur-containing amino acid involved in sulfur compound metabolism; its thiol group has a high affinity for heavy metals such as copper and zinc and reacts sensitively with ROS to neutralize free radicals [34]. Cys is also a major GSH precursor that affects intracellular GSH levels [35]. GSH in reduced and oxidized (GSSG) forms exist in cells as a non-protein thiol and is involved in oxidative stress regulation, detoxification, and neuroprotection [36]. In this study, the radical scavenging activities of Cys and GSH were confirmed (Table 1) and showed that they inhibited the accumulation of ROS and MDA by upregulating the expression of antioxidant enzymes in ethanol-treated ICR mice and HepG2 cells (Figure 4 and Figure 7). Consistent with our findings, L-theanine treatment was shown to restore GSH levels by increasing the activity of antioxidant enzymes in hepatocytes and attenuating ethanol-induced hepatotoxicity in mice [37]. Dietary polyphenols such as dihydromyricetin have also been documented to prevent ethanol-induced damage and improve ethanol-induced oxidative stress by regulating the expression of CYP2E1 and reducing ROS production [38].

Increased oxidative stress is mainly regulated by the activation of Nrf2, a transcription factor with anti-inflammatory and antioxidant properties [39]. Seitz et al. [40] showed that ethanol exposure upregulates Nrf2 as an adaptive response to CYP2E1-mediated oxidative stress. Therefore, the inhibition of oxidative stress through natural antioxidants via the Nrf2 pathway may help prevent alcoholic liver diseases. Quercetin and its derivatives activate the Nrf2 pathway and induce transcription of antioxidant enzymes in HepG2 cells, thereby restoring GSH depletion and suppressing ethanol-induced hepatocellular damage and inflammation [41]. Wang et al. [42] reported that anthocyanin-rich *Aronia melanocarpa* ameliorates chronic alcohol-induced Nrf2 downregulation, reduces antioxidant enzyme levels, and inhibits hepatic fat accumulation and inflammation in mice. In this study, mixed amino acids upregulated the expression of HO-1 through the activation of Nrf2 in HepG2 cells and effectively inhibited ethanol-induced oxidative stress (Figure 7 and Figure 8). The findings suggest that mixed amino acids exert antioxidant activity by activating the Nrf2 signaling pathway to inhibit ROS oxidative damage and prevent alcohol-induced liver diseases.

## 5. Conclusions

**Figure 9:**
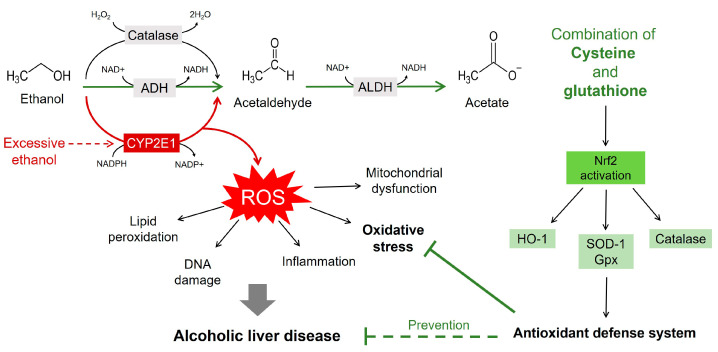
The main mechanisms of alcoholic liver damage and the mechanism of inhibition of alcoholic liver damage through inhibition of oxidative stress. The combination of cysteine and glutathione promotes ethanol metabolism and protects against alcohol-related liver damage through the activation of Nrf2. ADH, alcohol dehydrogenase; ALDH, aldehyde dehydrogenase; ROS, reactive oxygen species; Nrf2, nuclear factor erythroid 2–related factor 2; HO-1, heme oxygenase-1; GPx, glutathione peroxidase; SOD1, superoxide dismutase 1.

In this study, we demonstrated that the combination of Cys and GSH prevents ethanol-induced liver injury in ethanol-treated mice and HepG2 cells. In particular, mixing Cys and GSH at a 3:1 ratio (MIX) effectively improved hangover-related behaviors. MIX also promotes ethanol metabolism, promoting the removal of alcohol and acetaldehyde from the blood and inhibiting the release of AST and ALT from the liver. MIX contributed to the protective effect against alcoholic liver damage by suppressing oxidative stress through the activation of Nrf2 (Figure 9). These results suggest that MIX is an effective functional material in preventing liver damage caused by ethanol. However, since the results of this study were limited to the preventive aspect of ethanol-induced liver injury, further studies are needed to analyze the basic functionality of MIX and evaluate the potential of MIX as a therapeutic agent.

## Figures and Tables

**Table 1:** Antioxidant activity of chemicals using ABTS and DPPH radical scavenging assays.

Amino Acids	IC_50_ (mg/mL)	
	ABTS	DPPH
Arginine	10.47 ± 0.29 ^c^	35.42 ± 3.29 ^c^
Cysteine	0.05 ± 0.001 ^c^	0.05 ± 0.001 ^e^
Histidine	14.15 ± 0.36 ^c^	64.59 ± 4.38 ^b^
Taurine	227.38 ± 3.76 ^b^	84.18 ± 5.75 ^a^
Theanine	499.23 ± 14.20 ^a^	71.59 ± 1.28 ^b^
Glutathione	0.29 ± 0.01 ^c^	10.80 ± 0.75 ^d^

Values are expressed as mean ± SD (n = 3). Different letters indicate statistically significant differences at the *p* < 0.05 level (ANOVA followed by Tukey’s test). ABTS, 2,2′-Azino-bis-(3-ethylbenozothiazoline-6-sulfonic acid; DPPH, 2,2-diphenyl-1-picrylhydrazyl; IC_50_, the concentration of the chemicals required to reduce the absorbance of ABTS and DPPH radicals by 50%.

## Data Availability

Data is contained within the article.

## Supplementary Materials

The following supporting information can be downloaded at: https://www.mdpi.com/article/10.3390/antiox12101885/s1. Table S1: Antioxidant activity of amino acids using ABTS radical scavenging assay.

## References

[1] Contreras-Zentella M.L., Villalobos-Garcia D., Hernandez-Munoz R. (2022). Ethanol metabolism in the liver, the induction of oxidant stress, and the antioxidant defense system. Antioxidants.

[2] Hyun J., Han J., Lee C., Yoon M., Jung Y. (2021). Pathophysiological Aspects of Alcohol Metabolism in the Liver. Int. J. Mol. Sci..

[3] Yang Y.M., Cho Y.E., Hwang S. (2022). Crosstalk between oxidative stress and inflammatory liver injury in the pathogenesis of alcoholic liver disease. Int. J. Mol. Sci..

[4] Mackus M., Loo A.J.V., Garssen J., Kraneveld A.D., Scholey A., Verster J.C. (2020). The role of alcohol metabolism in the pathology of alcohol hangover. J. Clin. Med..

[5] Shiba S., Nakamoto N., Chu P.S., Ojiro K., Taniki N., Yamaguchi A., Morikawa R., Katayama T., Yoshida A., Aoki R. (2021). Acetaldehyde exposure underlies functional defects in monocytes induced by excessive alcohol consumption. Sci. Rep..

[6] Massart J., Begriche K., Hartman J.H., Fromenty B. (2022). Role of mitochondrial cytochrome P450 2E1 in healthy and diseased liver. Cells-Basel.

[7] Chen Y., Han M., Matsumoto A., Wang Y., Thompson D.C., Vasiliou V. (2018). Glutathione and Transsulfuration in Alcohol-Associated Tissue Injury and Carcinogenesis. Adv. Exp. Med. Biol..

[8] Tan H.K., Yates E., Lilly K., Dhanda A.D. (2020). Oxidative stress in alcohol-related liver disease. World J. Hepatol..

[9] Ferreira da Vinha A., Silva C.S., Costa C. (2023). Oxidative stress, antioxidants and biomarkers: Appreciation for analytical methods for health promotion. Int. Acad. Res. J. Intern. Med. Public. Health.

[10] Bellezza I., Giambanco I., Minelli A., Donato R. (2018). Nrf2-Keap1 signaling in oxidative and reductive stress. Biochim. Biophys. Acta Mol. Cell Res..

[11] Wang M., Zhu P., Jiang C., Ma L., Zhang Z., Zeng X. (2012). Preliminary characterization, antioxidant activity in vitro and hepatoprotective effect on acute alcohol-induced liver injury in mice of polysaccharides from the peduncles of *Hovenia dulcis*. Food Chem. Toxicol..

[12] Guo X., Li W., Xin Q., Ding H., Zhang C., Chang Y., Duan X. (2011). Vitamin C protective role for alcoholic liver disease in mice through regulating iron metabolism. Toxicol. Ind. Health.

[13] Eriksson C.J.P., Metsala M., Moykkynen T., Makisalo H., Karkkainen O., Palmen M., Salminen J.E., Kauhanen J. (2020). L-Cysteine Containing Vitamin Supplement Which Prevents or Alleviates Alcohol-related Hangover Symptoms: Nausea, Headache, Stress and Anxiety. Alcohol. Alcohol..

[14] Ozaras R., Tahan V., Aydin S., Uzun H., Kaya S., Senturk H. (2003). N-acetylcysteine attenuates alcohol-induced oxidative stress in the rat. World J. Gastroenterol..

[15] Yu M., Chen H., Liu P., Yang M., Zou L., Xiao D. (2020). Antioxidant Function and Metabolomics Study in Mice after Dietary Supplementation with Methionine. Biomed. Res. Int..

[16] Ramirez-Garcia O., Salinas-Moreno Y., Santillan-Fernandez A., Sumaya-Martínez M.T. (2022). Screening antioxidant capacity of Mexican maize (*Zea mays* L.) landraces with colored grain using ABTS, DPPH and FRAP methods. Cereal Res. Commun..

[17] You Y., Lee H., Chung C., Lee M.J., Jun W. (2016). Effect of mixture including hot water extract of *Houttuynia cordata* Thunb on ethanol-induced hangover in rats. J. Korean Soc. Food Sci. Nutr..

[18] Luong T.N., Carlisle H.J., Southwell A., Patterson P.H. (2011). Assessment of motor balance and coordination in mice using the balance beam. J. Vis. Exp..

[19] Walf A.A., Frye C.A. (2007). The use of the elevated plus maze as an assay of anxiety-related behavior in rodents. Nat. Protoc..

[20] Choi E.J., Kim H., Hong K.B., Suh H.J., Ahn Y. (2023). Hangover-Relieving Effect of Ginseng Berry Kombucha Fermented by Saccharomyces cerevisiae and Gluconobacter oxydans in Ethanol-Treated Cells and Mice Model. Antioxidants.

[21] Chung W.J., Chun H.J., Lee S.M. (2006). Socioeconomic Costs of Alcohol Drinking in Korea. J. Prev. Med. Public Health.

[22] Vonghia L., Leggio L., Ferrulli A., Bertini M., Gasbarrini G., Addolorato G., Group A.T.S. (2008). Acute alcohol intoxication. Eur. J. Intern. Med..

[23] Xu C., Xiong Q., Tian X., Liu W., Sun B., Ru Q., Shu X. (2022). Alcohol Exposure Induces Depressive and Anxiety-like Behaviors via Activating Ferroptosis in Mice. Int. J. Mol. Sci..

[24] King J.A., Nephew B.C., Choudhury A., Poirier G.L., Lim A., Mandrekar P. (2020). Chronic alcohol-induced liver injury correlates with memory deficits: Role for neuroinflammation. Alcohol.

[25] Zakhari S. (2006). Overview: How is alcohol metabolized by the body?. Alcohol. Res. Health.

[26] Guo R., Ren J. (2010). Alcohol and acetaldehyde in public health: From marvel to menace. Int. J. Environ. Res. Public. Health.

[27] Wall T.L., Luczak S.E., Hiller-Sturmhofel S. (2016). Biology, genetics, and environment: Underlying factors influencing alcohol metabolism. Alcohol. Res..

[28] Kwon H.J., Won Y.S., Park O., Chang B., Duryee M.J., Thiele G.E., Matsumoto A., Singh S., Abdelmegeed M.A., Song B.J. (2014). Aldehyde dehydrogenase 2 deficiency ameliorates alcoholic fatty liver but worsens liver inflammation and fibrosis in mice. Hepatology.

[29] Xiao C., Zhou F., Zhao M., Su G., Sun B. (2018). Chicken breast muscle hydrolysates ameliorate acute alcohol-induced liver injury in mice through alcohol dehydrogenase (ADH) activation and oxidative stress reduction. Food Funct..

[30] Gowda S., Desai P.B., Hull V.V., Math A.A., Vernekar S.N., Kulkarni S.S. (2009). A review on laboratory liver function tests. Pan Afr. Med. J..

[31] Jiang Y., Zhang T., Kusumanchi P., Han S., Yang Z., Liangpunsakul S. (2020). Alcohol Metabolizing Enzymes, Microsomal Ethanol Oxidizing System, Cytochrome P450 2E1, Catalase, and Aldehyde Dehydrogenase in Alcohol-Associated Liver Disease. Biomedicines.

[32] Lu Y., Wu D., Wang X., Ward S.C., Cederbaum A.I. (2010). Chronic alcohol-induced liver injury and oxidant stress are decreased in cytochrome P4502E1 knockout mice and restored in humanized cytochrome P4502E1 knock-in mice. Free Radic. Biol. Med..

[33] Li S., Tan H.Y., Wang N., Zhang Z.J., Lao L., Wong C.W., Feng Y. (2015). The role of oxidative stress and antioxidants in liver diseases. Int. J. Mol. Sci..

[34] Kim J.H., Jang H.-J., Cho W.-Y., Yeon S.-J., Lee C.-H. (2020). In vitro antioxidant actions of sulfur-containing amino acids. Arab. J. Chem..

[35] Atmaca G. (2004). Antioxidant effects of sulfur-containing amino acids. Yonsei Med. J..

[36] McBean G.J. (2017). Cysteine, glutathione, and thiol redox balance in astrocytes. Antioxidants.

[37] Li G., Ye Y., Kang J., Yao X., Zhang Y., Jiang W., Gao M., Dai Y., Xin Y., Wang Q. (2012). l-Theanine prevents alcoholic liver injury through enhancing the antioxidant capability of hepatocytes. Food Chem. Toxicol..

[38] Wang L., Oh J.Y., Yang H.W., Hyun J., Ahn G., Fu X., Xu J., Gao X., Cha S.H., Jeon Y.J. (2023). Protective effect of *Sargassum fusiforme* fucoidan against ethanol-induced oxidative damage in in vitro and in vivo models. Polymers.

[39] Yu S., Khor T.O., Cheung K.L., Li W., Wu T.Y., Huang Y., Foster B.A., Kan Y.W., Kong A.N. (2010). Nrf2 expression is regulated by epigenetic mechanisms in prostate cancer of TRAMP mice. PLoS ONE.

[40] Seitz H.K., Bataller R., Cortez-Pinto H., Gao B., Gual A., Lackner C., Mathurin P., Mueller S., Szabo G., Tsukamoto H. (2018). Alcoholic liver disease. Nat. Rev. Dis. Primers.

[41] Lee S., Lee J., Lee H., Sung J. (2019). Relative protective activities of quercetin, quercetin-3-glucoside, and rutin in alcohol-induced liver injury. J. Food Biochem..

[42] Wang Z., Liu Y., Zhao X., Liu S., Liu Y., Wang D. (2020). *Aronia melanocarpa* prevents alcohol-induced chronic liver Injury via regulation of Nrf2 signaling in C57BL/6 mice. Oxid. Med. Cell Longev..

