# Supplementary material for: Combination of Cysteine and Glutathione Prevents Ethanol-Induced Hangover and Liver Damage by Modulation of Nrf2 Signaling in HepG2 Cells and Mice
Source: Antioxidants (Basel). 2023 Oct 20;12(10):1885. doi: 10.3390/antiox12101885 (PMC10604027; doi:10.3390/antiox12101885)
Supplement: Supplementary file 1 [file antioxidants-12-01885-s001.zip › antioxidants-2652045-supplementary.pdf]

Table S1. Antioxidant activity of amino acids using ABTS radical scavenging assay

Amino acids	Antioxidant activity (μg AEAC/mg of sample)
Asparagine	0.14 ± 0.01
Alanine	0.09 ± 0.02
Arginine	4.43 ± 0.07
Aspartic acid	0.12 ± 0.08
Cysteine	870.84 ± 9.20
Glutamine	0.02 ± 0.03
Glutamic acid	0.01 ± 0.09
Glycine	0.08 ± 0.04
Histidine	3.81 ± 0.05
Isoleucine	0.76 ± 0.02
Leucine	0.52 ± 0.04
Methionine	0.14 ± 0.02
Serine	0.09 ± 0.04
Tryptophan	1.45 ± 0.08
Tyrosine	0.20 ± 0.09
Valine	0.58 ± 0.03

Values are expressed as mean ± SD (n = 3). AEAC, ascorbic acid equivalent antioxidant capacity;

ABTS, 2,2'-azino-bis (3-ethylbenozothiazoline-6-sulfonic acid)
